# Postpartum eruption of enoxaparin‐induced erythema multiforme

**DOI:** 10.1002/ccr3.1752

**Published:** 2018-08-23

**Authors:** Eric D. Schadler, Joel C. Joyce, Reshma N. Haugen

**Affiliations:** ^1^ University of Chicago Pritzker School of Medicine Chicago Illinois; ^2^ Division of Dermatology NorthShore University HealthSystem Skokie Illinois

**Keywords:** drug eruption, drug reaction, enoxaparin, erythema multiforme, low molecular weight heparin

## Abstract

Enoxaparin is a commonly used hospital medication and in rare instances may result in development of erythema multiforme. Management of these patients can be challenging. Physicians must maintain a high index of suspicion and consider the indication for enoxaparin therapy prior to withdrawal of the medication.

## 1 INTRODUCTION

Erythema multiforme (EM) is a cutaneous condition that manifests with variable phenotypes, the hallmark lesion being targetoid in nature. While commonly attributed to infections, EM can also be secondary to drugs and malignancy; however, the causative etiology often remains unknown. Enoxaparin is a frequently used anticoagulant, often administered following surgery or during pregnancy to prevent deep vein thrombosis. Herein, we describe a rare cutaneous drug reaction to enoxaparin resulting in erythema multiforme.

## 2 CASE REPORT

**Figure 1:**
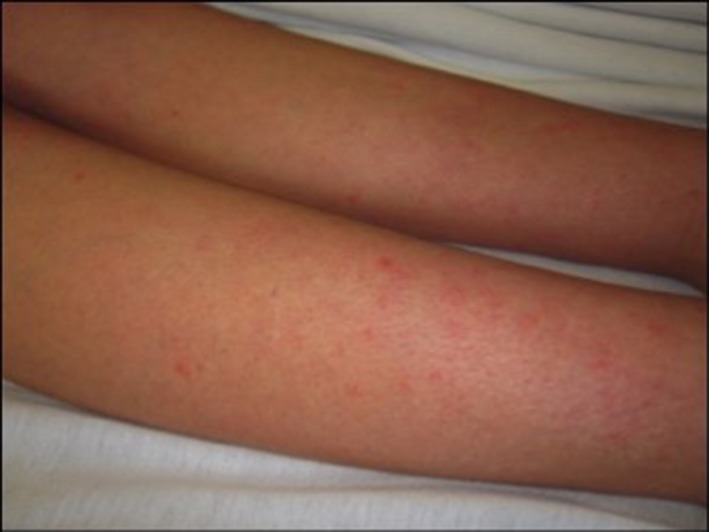
Pink macules and papules on the legs were present at the initial presentation

A 27‐year‐old postpartum woman presented to the emergency room with pruritic pink macules and papules distributed on the bilateral legs, forearms, shoulders, and abdomen (Figure 1). Two weeks prior to presentation she was diagnosed with a hospital‐acquired pulmonary embolus following preterm delivery of her twin pregnancy. She was immediately started on enoxaparin (50 mg) administered daily following identification of the embolus. After approximately 10 days of therapy she first noticed the pruritic lesions beginning.

She denied any new food, medication, or other exposures with the exception of enoxaparin (50 mg daily). Review of systems was negative for fevers, chills, sick contacts, sores on the mucus membranes, abdominal pain, or other systemic complaints. No viral sampling was performed given the lack of systemic symptoms. Laboratory work‐up for heparin/platelet factor‐4 antibodies was negative and complete blood count was unremarkable (WBC = 3.9 × 10^3^/μL; RBC = 3.74 × 10^6^/μL; Hemoglobin = 11.2 g/dL; platelet count = 246 × 10^3^/μL). She was diagnosed with a nonspecific inflammatory papular dermatitis and started on triamcinolone ointment with planned outpatient follow‐up.

**Figure 2:**
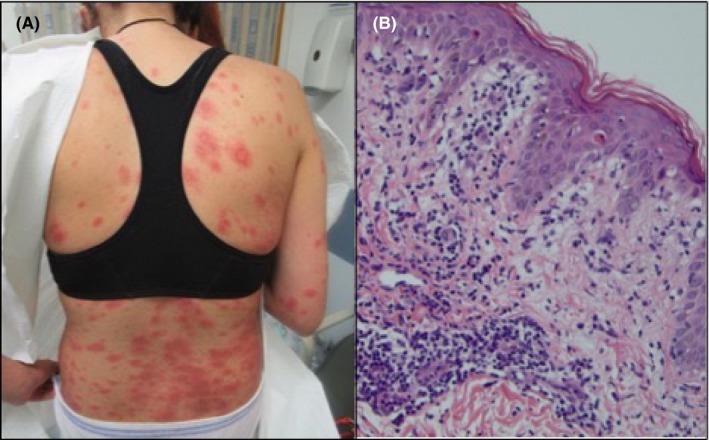
A, The rash evolved into numerous papules with surrounding erythema. B, H&E stain at 20× magnification was consistent with a drug eruption

Three days after evaluation in the emergency department she presented to the outpatient dermatology clinic for progression of her rash and significant pruritus. A review of systems was unchanged. Physical examination revealed dozens of 2‐8‐mm blanching pink papules with surrounding faint halos distributed on the arms, legs, abdomen, and back (Figure 2A). Due to the change in morphology, biopsies of an active abdominal lesion for H&E stain and immunofluorescence were performed to rule out pregnancy‐related dermatoses including late onset pruritic urticarial papules and plaques of pregnancy (PUPPP) and pemphigoid gestationis. In the meantime, she was continued on triamcinolone.

**Figure 3:**
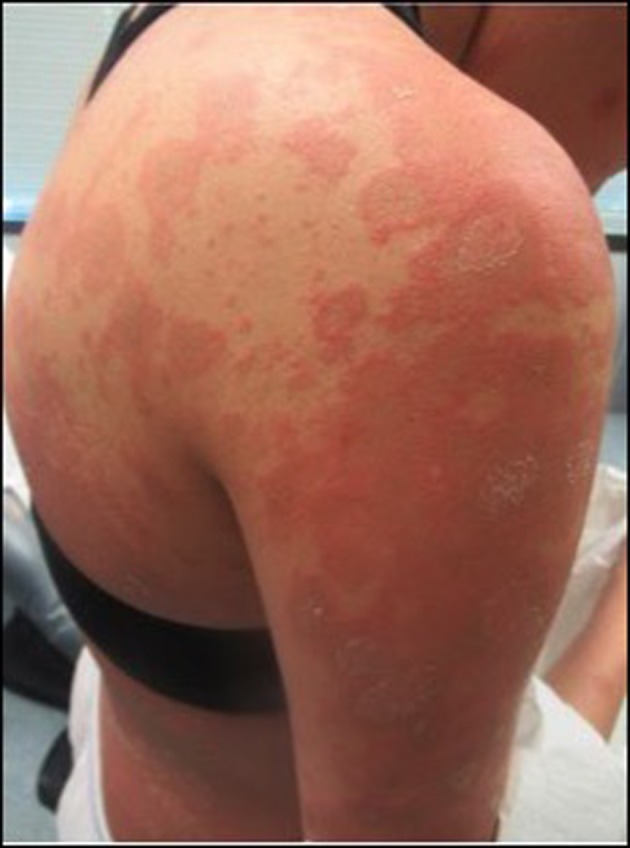
Examination revealed characteristic targetoid lesions

She returned to the clinic 1 week following biopsy for follow‐up. During this time period her condition had considerably worsened necessitating an urgent care visit where she was started on 20 mg of prednisone per day, with minimal improvement after 4 days of treatment. On physical examination, the development of numerous targetoid pink plaques with dusky centers and erosions, distributed on the arms, legs, back, abdomen, and chest, was seen (Figure 3). No mucosal involvement or systemic symptoms were present except extreme pruritus. Histologic findings revealed perivascular lymphocytic inflammation, necrotic keratinocytes, and increased dermal mucin consistent with a drug eruption (Figure 2B). Interestingly, an eosinophilic infiltrate was not identified as would be expected in a drug‐related process. Direct immunofluorescence was negative. Based on the temporal relation of lesions to initiation of enoxaparin, a diagnosis of enoxaparin‐induced erythema multiforme was made. She was started on prednisone 60 mg daily and initiated warfarin therapy for anticoagulation. Enoxaparin was continued as bridging therapy until achieving therapeutic anticoagulation with warfarin, and subsequently discontinued.

**Figure 4:**
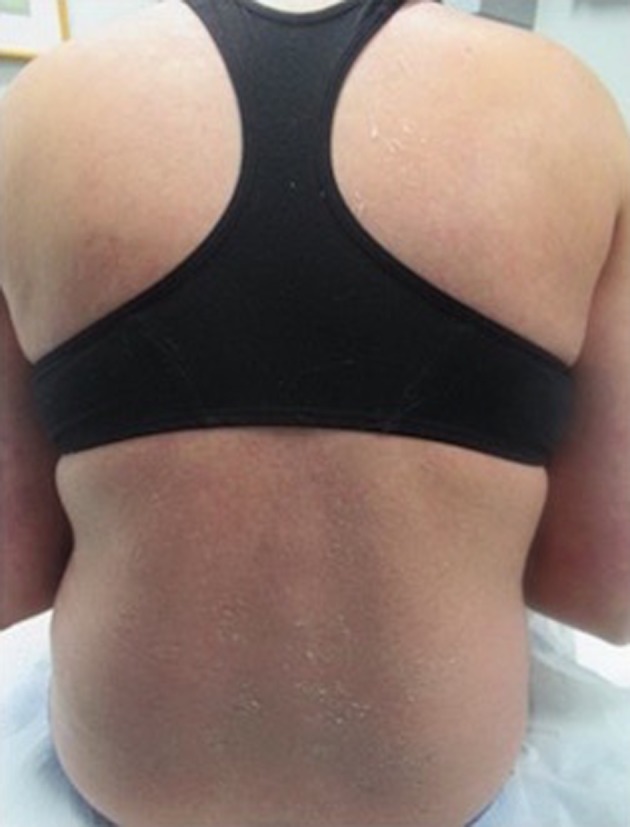
Following discontinuation of enoxaparin, lesions began to desquamate and no new lesions were identified

Upon discontinuation of enoxaparin there was rapid improvement within a week with cessation of new lesion development and desquamation of old lesions (Figure 4).

## 3 DISCUSSION

Adverse cutaneous drug reactions (ACDR) occur in approximately 1%‐3% of hospitalized patients.^1^ Enoxaparin, a low‐molecular weight heparin (LMWH), is a commonly used medication in the inpatient setting. It functions by binding antithrombin III, thereby accelerating the inactivation of factor X_a_ within the coagulation cascade. The most common cutaneous reaction to enoxaparin is delayed‐type hypersensitivity reaction at the injection site. A less common but recognized risk is heparin‐induced thrombocytopenia resulting from antibodies against heparin/platelet factor‐4. Rarely, reports of bullous hemorrhagic dermatosis and drug reaction with eosinophilia and systemic symptoms syndrome are described.^2^, ^3^ To our knowledge no cases of enoxaparin‐induced erythema multiforme have been described.

Erythema multiforme is an acute mucocutaneous condition that classically presents with targetoid lesions. It is most commonly secondary to a recent viral infection (>90%); however, other causes include drugs, autoimmunity, malignancy, and immunizations.^4^ Currently, the pathogenesis is not fully understood, and most studies that discuss this use cases of Herpes simplex virus (HSV)‐induced EM. Interestingly, HSV‐induced EM involves interferon‐γ as a principle cytokine whereas drug‐induced EM is associated with tumor necrosis factor alpha (TNF‐α) implying a different disease pathway.^4^ Although biopsy is not required for diagnosis of EM, it may be useful in atypical cases to rule out other causes. This case of drug‐induced EM was unique for two distinct reasons. First, management of her condition created a clinical conundrum. Standard management of drug‐induced EM includes immediate removal of the offending agent. This patient had multiple risk factors for thrombosis and development of pulmonary emboli, thus prohibiting immediate withdrawal of enoxaparin. In the setting of her recent hospital‐acquired pulmonary embolus, the decision to initiate warfarin therapy and bridge with enoxaparin until achieving a therapeutic international normalized ratio (INR) was made. This decision was dictated by the maternal‐fetal‐medicine team responsible for management of her anticoagulation; it is unclear if the use of a different LMWH was discussed. Alternative options to use a heparin bridge and immediately discontinue enoxaparin were discussed, but would necessitate hospital admission making this cost ineffective and placing the patient at additional hospital associated risks. Until enoxaparin could be withdrawn, high‐dose prednisone provided temporary symptomatic relief.

Second, this case was diagnostically challenging due to our patient's postpartum presentation. New‐onset dermatoses in the postpartum period may include a variety of pregnancy‐related conditions, including pruritic urticarial papules and plaques of pregnancy (PUPPP), papular dermatitis of pregnancy, and pemphigoid gestationis. Although the rash in this case was extending further into the postpartum period than expected, PUPPP was considered given the bland appearance and the extreme pruritus associated with her initial presentation. Once the clinical morphology changed our differential diagnosis shifted and biopsy was performed. Until this point, clinical suspicion for EM or drug‐related process was low.

## 4 CONCLUSION

This case illustrates a rare drug reaction to enoxaparin. Diagnosis of EM demands a high index of suspicion as it may resemble multiple conditions. Consideration of drug‐related dermatoses can decrease time to diagnosis and allow for rapid withdrawal of an offending agent.

## CONFLICTS OF INTEREST

None to disclose.

## AUTHOR CONTRIBUTIONS

ES: Drafted the initial manuscript and assisted with revision process. Collected photographs and data to be used in manuscript. JJ: Performed major revisions of manuscript for important intellectual content. Assisted with gathering photographs. RH: Conception of the case report. Performed major revisions of manuscript for important intellectual content.
